# Depression and anxiety in patients with different rare chronic diseases: A cross-sectional study

**DOI:** 10.1371/journal.pone.0211343

**Published:** 2019-02-20

**Authors:** Natalie Uhlenbusch, Bernd Löwe, Martin Härter, Christoph Schramm, Christina Weiler-Normann, Miriam K. Depping

**Affiliations:** 1 Department of Psychosomatic Medicine and Psychotherapy, University Medical Center Hamburg-Eppendorf, Hamburg, Germany; 2 Department of Medical Psychology, University Medical Center Hamburg-Eppendorf, Hamburg, Germany; 3 1st Department of Medicine, University Medical Center Hamburg-Eppendorf, Hamburg, Germany; 4 Martin Zeitz Center for Rare Diseases, University Medical Center Hamburg-Eppendorf, Hamburg, Germany; Chiba Daigaku, JAPAN

## Abstract

**Objective:**

Empirical evidence on depression and anxiety in patients with rare diseases is scarce but can help improve comprehensive treatment. The objectives of this study were to investigate the frequency of depression and anxiety in this heterogeneous population and to examine aspects associated with increased psychopathology.

**Methods:**

*N* = 300 patients with 79 different rare diseases (female:80%, age:*M* = 44.3(12.8), range:16–74 years) participated in a cross-sectional online study. We determined the percentages of patients reporting elevated depression (PHQ-9) and anxiety (GAD-7) scores. We calculated two linear regressions with depression and anxiety as outcomes. Predictor variables were diagnosis-related aspects (diagnosis assigned to ICD-10 chapter, visibility of symptoms, time since diagnosis, comorbid diseases), perceived somatic-symptom-severity (PHQ-15), illness-perceptions (consequences, control, identity, concern, understanding and treatment control; B-IPQ-R), coping mechanisms (constructive attitudes, active engagement in life) and social support (heiQ). We controlled for gender, age and depression or anxiety depending on the outcome.

**Results:**

42% of the patients (95%CI [36.41%,47.59%]) reported depression scores indicating moderately or severely elevated symptom levels. Regarding anxiety, this applies to 23% (95%CI [18.54%,28.06%]). Variables significantly associated with depression were higher perceived somatic-symptom-severity (*B* = 0.41,*p* < .001), less control (*B* = .17,*p* < .05), lower levels of concern (*B* = -0.32,*p* < .01) and less constructive attitudes (*B* = -1.40,*p* < .001). No diagnosis-related variables were associated with depression. Variables significantly associated with anxiety were diseases of the circulatory system compared to congenital malformations (*B* = 1.88,*p* < .05), less consequences (*B* = -0.32,p < .05) and more concern (*B* = -0.32,*p* < .01).

**Conclusion:**

The data reveal first insights into depression and anxiety in patients with different rare diseases. High percentages of patients showed clinically relevant symptom burden. No diagnosis-related differences were found in depression while anxiety seems to be particularly frequent in patients with rare diseases of the circulatory system. Besides perceived somatic symptom severity, cognitive appraisal seems to be linked to depression. Supporting patients in coping with their disease may help reduce psychopathology and therefore improve overall health.

## Introduction

Rare diseases are a group of diseases defined by a low prevalence (<1:2000) and characterized by a great heterogeneity [1]. Worldwide, there are around 7000 different rare diseases varying tremendously in their clinical appearance, course and etiology. In spite of the heterogeneity of rare conditions, there may also be shared burdens among different rare diseases. The majority of rare diseases are complex, chronic, progressive, degenerative, often life-threatening and go hand in hand with reduced quality of life [1]. In addition, access to adequate care is often limited and information about the diseases is often sparse due to the rarity of each condition [1]. Furthermore, delay in diagnosis often results in negative experiences such as frustration and self-doubt [2]. Another shared experience among patients with different rare diseases may be the psychopathological burden since chronic illness, in general, is often associated with depression and anxiety [3]. Scientific publications on psychopathology in patients with rare diseases are scarce. However, reports of stakeholders such as patient organizations or pharmaceutical companies indicate high psychological burden of rare diseases and increased psychopathology levels [4, 5]. Some further studies showed increased levels of depression and anxiety in patients with specific rare diseases, for instance in pulmonary arterial hypertension [6] or Marfan syndrome [7], whereas a few studies did not, for instance in patients with hereditary primary immunodeficiency [8]. The majority of those studies are either case reports or quantitative studies with small sample sizes focusing on one specific rare disease. In conclusion, current empirical research about depressive and anxious syndromes in patients with rare diseases is limited. In the absence of a medical cure for the majority of rare diseases [1], targeting depression and anxiety in treatment can be critical for the quality of life in this group of patients. Further research is therefore needed to better understand depression and anxiety in this patient population in order to improve overall health.

Depression and anxiety in more common chronic physical conditions such as coronary heart disease or diabetes can be associated with increased mortality [9] as well as poor prognosis of the physical disease [10] and have an impact on medical symptom burden [11] and on health behaviors such as compliance [12]. Additionally, depression is associated with an increase of about 50% in costs of chronic medical illness [11]. Therefore, detecting and addressing depression and anxiety in chronically ill individuals can improve outcomes beyond mental health. Understanding the conditions under which the symptoms emerge or, vice versa, under which conditions patients adjust well to the chronic condition can help to provide adequate, targeted treatment and prevention.

Whether or not individuals adjust well to their chronic condition and stay mentally healthy is affected by many different aspects within the individuals as well as in their social environment. Variables associated with poor adjustment in more frequent chronic conditions are for instance pessimistic attributional styles, behavioral avoidance, rumination or dysfunctional social interactions such as social constrains, conflicts or overprotective behavior of spouses [13]. On the other hand, variables associated with good adjustment to a chronic illness include social support, positive behavioral coping-mechanisms (e.g. information gathering or problem solving) and cognitive coping-mechanisms (such as optimistic and constructive attributional styles) as well as personality traits such as self-esteem [13]. Moreover, illness perceptions impact adjustment to chronic conditions [14]. For instance, control beliefs (e.g. the perceived ability and resources to manage pain) positively impact adjustment [15, 16], whereas a high perceived extent of the illness affecting one’s life is a risk factor for elevated levels of psychopathology in common chronic diseases [17]. With regard to rare diseases, to our knowledge, there are currently no studies examining underlying mechanisms or protective and risk factors for depression and anxiety.

To date, it is unclear whether the aforementioned evidence on risk and protective factors for depression and anxiety in more common chronic diseases is applicable to rare chronic diseases. Rare and more common chronic conditions go along with some mutual burdens such as persisting symptoms, high symptom severity, and reduced quality of life [18]. Thus, the prevalence of psychopathology and the conditions under which it occurs may also be similar. However, individuals with rare diseases may face some additional burdens due to the rarity of each condition, such as lack of knowledge about the disease, lack of access to specialized medical care and wandering contact to peers with the same diagnosis [19]. Moreover, from a health care perspective, physicians describe treatment of rare diseases as more complex compared to more common diseases [5]. It remains unclear if these differences revolve in different patterns with regard to psychopathology or if knowledge about depression and anxiety in more common conditions is applicable to the field of rare diseases.

One reason why depression and anxiety in the field of rare diseases is not well understood may be that research on rare diseases is generally challenging due to several barriers, such as the difficulty of achieving sufficiently large sample sizes (for a more detailed description of the challenges in investigating rare diseases see IQWiG, 2014 [20]). One approach to overcome these challenges could be to examine different rare diseases concurrently, since patients with heterogeneous rare diseases face shared burdens [2]. Investigating patients with rare diseases across different diagnoses enables research on this patient population methodologically by allowing for sample sizes that can be subject to statistical analysis. Further, the approach can help address potential shared treatment needs of patients across rare disease diagnoses. Identifying shared treatment needs can help better treat this group of patients in the face of finite health care resources. The need for studies investigating different rare diseases concurrently from a psychological perspective has recently been stressed [19]. Despite the promise a perspective across diagnoses may have, diagnosis-related differences could also lead to different patterns with regard to the development of psychopathological symptoms. In more common chronic diseases, some studies point in the direction that diagnosis-related patterns impact psychopathology outcomes [3, 16]. It is unclear whether diagnosis-related aspects affect depression and anxiety in rare chronic diseases.

Based on the considerations above, objectives of the current study were primarily to investigate the frequency of depression and anxiety in patients with different rare diseases and identify aspects associated with depression and anxiety symptoms. In doing so, we took into account variables, which are relevant in the development of depression and anxiety in more common chronic conditions. These include perceived somatic symptom severity, illness perceptions, social support and coping mechanisms. To the best of our knowledge, this is the first study investigating depression and anxiety in patients with different rare diseases concurrently. To examine if this approach is valid, we further aimed to investigate if the diagnosis and other diagnosis-related characteristics of the different rare diseases are associated with depression and anxiety.

## Materials and methods

### Procedure/ Study design

The present study was a cross-sectional online study. It is part of the project ‘Patients for patients: qualified peer counseling and self-management for patients with rare chronic diseases’, which aims to develop a new program to improve disease management in patients with rare diseases.

### Participants

We recruited participants through centers for rare diseases, patient organizations and self-help groups for rare diseases across Germany and specialized outpatient clinics for rare diseases at the University Medical Center Hamburg-Eppendorf. Information about the study and a link to the online survey was disseminated on websites as well as with printed flyers and via mailing lists of patient organizations. Patients interested in participating could contact the research team in case of any questions or directly participate anonymously in the study by following the link. Before conducting the online survey, participants gave informed consent online. No written consent was required since data were collected and analyzed anonymously. Inclusion criteria were the diagnosis of any rare disease and an age of at least 16 years. Exclusion criteria were an unclear diagnosis as well as if the disease was not considered rare or if patients reported that the diagnosis was not given by a physician. Rarity was defined as not occurring in more than one in 2000 individuals [1]. If information about the prevalence was not available, a disease was included if it was listed in the orpha.net register of rare diseases (https://www.orpha.net/consor/cgi-bin/Disease.php?lng=EN). We further excluded participants who completed less than 85% of the survey or for whom the primary outcomes (depression and anxiety) were not calculable due to missing values. Ethics approval was given through the independent ethics committee of the Hamburg Medical Chamber (Ärztekammer Hamburg) on February 2, 2016 (reference number PV5088).

### Measures

#### Depression and anxiety

Primary outcomes of this study were symptoms of depression and anxiety. We measured depression using the German version of the depression module of the Patient Health Questionnaire-9 (PHQ-9), a 9-item screening instrument determining severity levels of depressive symptomatology [21–24]. Sum-score cut-off values of 5, 10, 15 and 20 represent thresholds for mild, moderate, moderately severe, and severe depression, respectively. As a single cut-off value indicating the occurrence of a major depression, the authors recommend a score of 10 or higher [21]. The instrument demonstrates excellent internal consistency (α = .89), excellent test-retest reliability as well as criterion and construct validity [25].

Anxiety was assessed with the German version of the Generalized Anxiety Disorder 7-item scale (GAD-7) [26, 27]. A sum-score value of ≥10 was identified as ideal cut-off point indicating generalized anxiety disorder [26]. Although it has the best operating characteristics in detecting generalized anxiety disorder, the instrument has shown to be a useful screening tool for any other anxiety disorder [28]. The GAD-7 demonstrates excellent internal consistency (α = .92), good test-retest reliability (intraclass correlations = .83) as well as criterion, construct, factorial, and procedural validity [26].

#### Diagnosis-related characteristics

Diagnosis was self-reported. Further, we asked patients to report who gave the diagnosis. In research on more common chronic conditions, different diseases are often compared directly. Since we included patients with rare diseases, we expected the subgroups of each disease to be too small to be analyzed. We built disease sub-categories according to the affiliation to ICD-10 (10th revision of the International Statistical Classification of Diseases and Related Health Problems) chapters. For instance, pulmonary arterial hypertension was assigned to diseases of the circulatory system while primary sclerosing cholangitis was assigned to diseases of the digestive system. This approach was chosen since the assignment to ICD-10 chapters offers an internationally accepted, valid, standardized and objective way to categorize different diseases. The ICD-10 classification is based on etiology or affected organ system. There are studies on more common chronic conditions indicating that the affected organ system can lead to differences in psychopathology [3, 29, 30]. Another diagnosis-related characteristic was the visibility of symptoms. We further controlled for the time since diagnosis was given and whether patients have comorbid diseases. All diagnosis-related characteristics were self-reported.

#### Perceived somatic symptom severity

We measured perceived somatic symptom severity using the German version of the PHQ-15 [31, 32]. The instrument assesses 15 somatic symptoms; each symptom scored from 0 (“not bothered at all”) to 2 (“bothered a lot”). It is a well-validated and reliable measure for assessing the perceived burden of somatic symptoms and screening for somatoform disorders [33].

#### Illness perceptions

Different dimensions of illness perceptions were assessed with the German brief version of the illness perception questionnaire (Brief IPQ-R) [34]: consequences (the extent of the illness affecting one’s life), personal control (the perceived control one has over the illness), identity (the extent one experiences symptoms of their illness), concern (the extent one is worried about their disease), understanding (the extent one understands their illness) and treatment control (the extent to which the patient believes that the treatment is helpful). The instrument uses single items to assess each dimension on a scale from 0 to 10. It has been shown to be a valid and reliable measure of illness perceptions in a variety of illness groups. It demonstrates good psychometric properties including concurrent, predictive and discriminant validity as well as good re-test reliability [34].

#### Social support and coping mechanisms

Subscales of the Health Education Impact Questionnaire (heiQ) [35] were used in order to assess social support as well as coping mechanisms on a cognitive (constructive attitudes) and behavioral (active engagement in life) level. The heiQ is an instrument originally designed to evaluate patient education and self-management interventions. It consists of eight subscales measuring coping skills, which are often targeted in self-management interventions. In this study, we used three subscales: social integration and support, constructive attitudes and approaches, and positive and active engagement in life. The evaluation study indicates that all the heiQ scales have either good or excellent psychometric properties [35].

### Statistical analyses

We calculated Cronbach’s Alpha in order to determine internal consistencies of all scales and subscales used in the study. For the main analyses, we calculated means, standard deviations and 95% confidence intervals for the primary outcomes depression (PHQ-9) and anxiety (GAD-7). We determined percentages of patients above the cut-off-value in the PHQ-9 (≥10) and GAD-7 (≥10). T-tests for one sample were used to compare PHQ-9 and GAD-7 means in our sample with the norm samples. We used T-tests for independent samples to investigate gender-related differences in depression and anxiety within our sample.

We further calculated two multiple linear regression models with either depression or anxiety as the dependent variable and potential correlates of depression and anxiety as predictors. We statistically controlled for gender as well as for anxiety when depression was the outcome and vice versa. In order to investigate if the diagnosis and other diagnosis-related aspects of different rare diseases account for variation in depression and anxiety, the regression model included different blocks: control variables (gender, age and either depression or anxiety), diagnosis-related characteristics (ICD-10 categories, visibility of the disease, time since diagnosis, comorbid diseases) and psychosocial aspects (perceived somatic symptom severity, consequences, personal control, identity, concern, understanding, treatment control, social integration and support, constructive attitudes and approaches, positive and active engagement in life). As a categorical variable, ICD-10 chapter was transformed into several dummy variables. With this approach, one reference group is compared with every other group. Since we were primarily interested in the overall effect of the categorical variable ICD-10-chapter we refrained from making additional pairwise comparisons. Based on recommendations [36, 37] we chose the largest subgroup as reference group. Categories with less than 10 cases were excluded for this analysis. Assumptions for regression analyses were checked before interpreting the results. Normal distribution of residuals as well as homoscedasticity were examined graphically using normality plots of residuals as well as scatterplots. Durbin-Watson-Test was used in order to check if residuals were auto-correlated. In case any assumptions were violated we considered calculating bootstrap confidence intervals as a robust regression measure following the recommendations of Field (2013) [36].

In order to estimate the required sample size for performing the regression analysis, we conducted a power calculation using G*Power 3.1.9.2. Conservatively assuming small effect sizes (*f*^*2*^ = 0.15), a minimum sample size of *N* = 230 is required with a 0.05 level of significance and an actual power of 95% in order to perform multiple linear regression analysis with 22 predictors.

## Results

### Preliminary analyses

#### Sample characteristics

**Fig 1:**
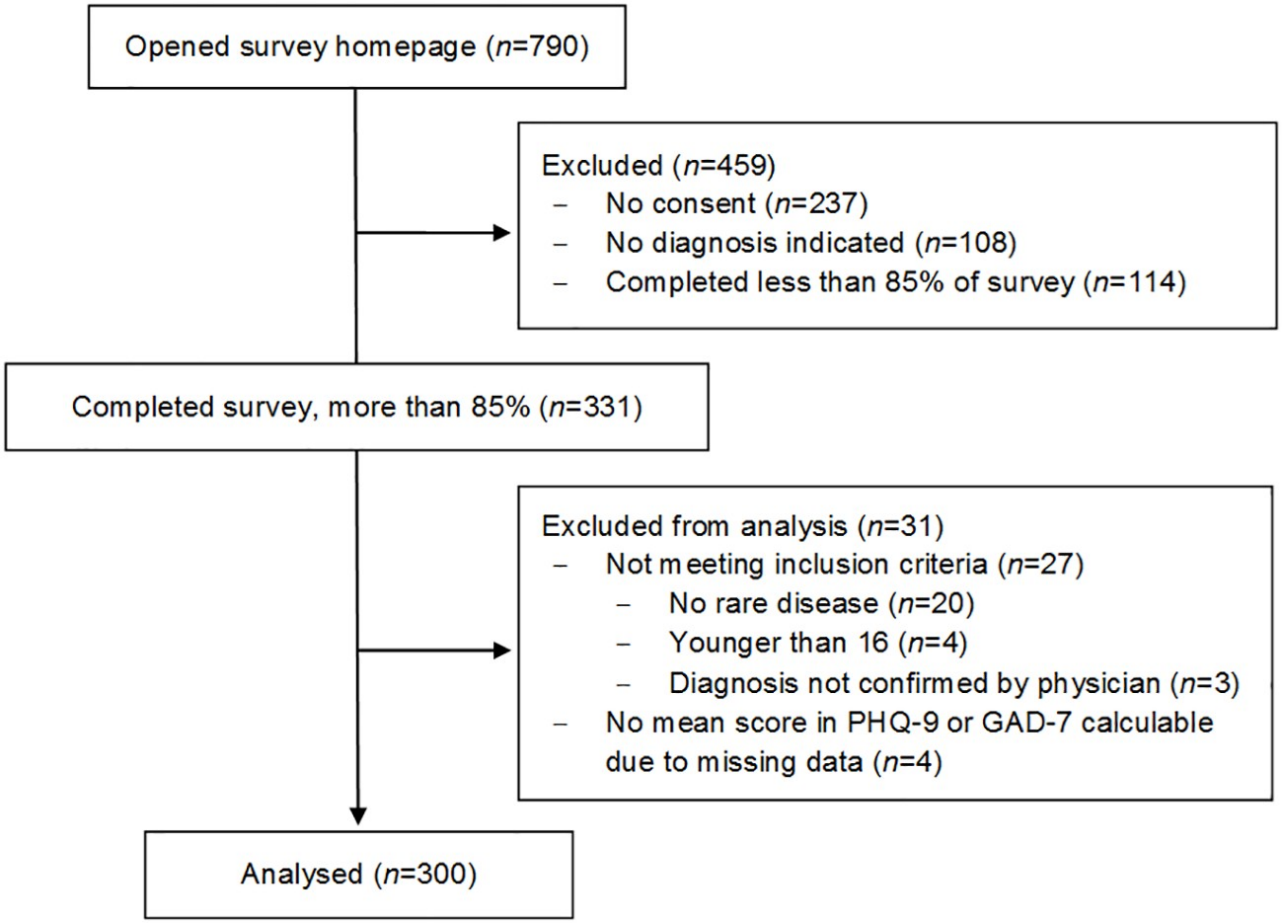
Flowchart.

Out of 790 individuals that opened the online survey, *N* = 331 participants completed it, which represents a completion rate of approximately 42%. *N* = 31 participants were excluded from analyses, resulting in a sample size of *N* = 300 participants. Fig 1 displays participant flow over the course of recruitment in detail.

The majority of the participants were female (82%, *n* = 245). Mean age was 44.3 years (*SD* = 12.8, range 16–74). Almost half of the patients (*n* = 139) reported to be either self-employed or employed and 8% (*n* = 25) were students. About half of the patients (*n* = 138) were married, 35% (*n* = 104) were single and the rest either separated, divorced, widowed or other. 95% had German citizenship. A total of 79 different rare diseases were represented among participants, with neurofibromatosis (*n* = 37), primary biliary cholangitis (*n* = 26), autoimmune hepatitis (*n* = 25), pulmonary arterial hypertension (*n* = 22), Marfan syndrome (*n* = 19) and primary sclerosing cholangitis (*n* = 16) being the largest subgroups. Table 1 shows all rare diseases within the sample, categorized into ICD-10 chapters as well as the number of patients assigned to each category. Two participants could not be assigned to a category due to being affected by several rare diseases.

**Table 1:** Diagnoses assigned to ICD-10 chapters.

ICD-10 category	Assigned diagnoses	*n*
A00-B99—Certain infectious and parasitic diseases	Yersiniosis	1
C00-D48—Neoplasms	hairy cell leukemia, multiple endocrine neoplasia, chordoma, essential thrombocythemia, paraganglioma, osteomyelofibrosis, lymphangioleiomyomatosis	10
D50-D90—Diseases of the blood and blood-forming organs and certain disorders involving the immune mechanism	ADA deficiency, chronic septic granulomatosis, autoimmune hemolytic anemia, hereditary angioedema	4
E00-E90—Endocrine, nutritional and metabolic diseases	Addison disease, lysosomal storage disease, Cushing syndrome, hypopituitarism, Fabry disease, diabetes insipidus, adrenomyeloneuropathy, porphyria, acromegaly, amyloidosis, alpha-1-antitrypsin deficiency, autoimmune polyglandular failure	29
G00-G99—Diseases of the nervous system	syringomyelia, periodic paralysis, neuromyelitis optica, alternating hemiplegia of childhood, cerebellar ataxia, acute disseminated encephalomyelitis (ADEM), myelitis, intracranial hypertension, primary myopathy, dystonia, chronic inflammatory demyelinating polyneuropathy (CIDP), lymphocytic hypophysitis, hereditary spastic paraplegia, stiff person syndrome	32
H00-H59—Diseases of the eye and adnexa	Fuchs heterochromic iridocyclitis	1
I00-I99—Diseases of the circulatory system	pulmonary arterial hypertension (PAH), other secondary pulmonary hypertension, mitral valve prolapse, erythromelalgia, long QT syndrome	28
J00-J99—Diseases of the respiratory system	idiopathic pulmonary fibrosis (IPF)	1
K00-K93—Diseases of the digestive system	primary sclerosing cholangitis (PSC), primary biliary cholangitis (PBC), autoimmune hepatitis (AIH), overlap syndrome	73
L00-L99—Diseases of the skin and subcutaneous tissue	pemphigus, collagenosis, autoinflammatory disease, vasculitis	13
M00-M99—Diseases of the musculoskeletal system and connective tissue	Wegener granulomatosis, polymyositis, scleroderma, lupus erythematosus (SLE), microscopic polyangiitis, scleredema adultorum Buschke	13
N00-N99—Diseases of the genitourinary system	glomerulonephritis	1
Q00-Q99—Congenital malformations, deformations and chromosomal abnormalities	Marfan syndrome, neurofibromatosis type 1 (NF1), neurofibromatosis type 2 (NF2), schwannomatosis, thoracic aortic aneurysm, polycystic liver disease, Loeys-Dietz syndrome, Stickler syndrome, nail-patella syndrome, cavernoma, single ventricle, trichorhinophalangeal syndrome, aortic stenosis, tetralogy of Fallot, transposition of the great arteries, congenital malformation of heart unspecified, ventricular septal defect (VSD), atrial septa defect (ASD), complex congenital heart defect	92
No assignment possible due to several rare diagnoses		2

Signs of the rare disease were visible in 44% (*n* = 131) of the patients. Besides the rare disease, almost 70% (*n* = 207) of the participants had further diseases. The average time since participants received the diagnosis was 150 months (*SD* = 137.8). 20% (*n* = 60) of the patients reported to currently receive psychotherapeutic treatment and one third (*n* = 100) patients had psychotherapy in the past.

### Internal consistencies

Cronbach’s Alpha as a measure of internal consistency reached from good to excellent for all scales and subscales used in the analysis (PHQ-9: α = .86; GAD-7: α = .91; PHQ-15: α = .79; heiQ: positive and active engagement in life: α = .85, constructive attitudes and approaches: α = .89, social integration and support: α = .90).

### Symptoms of depression and anxiety

The average depression score was *M* = 9.10 (*SD* = 5.98, 95%CI [8.24, 9.78]). The mean score in anxiety was *M* = 6.51 (*SD* = 5.38, 95% CI [5.90, 7.12]). 42% of the patients (95%CI [36.41%, 47.59%]) showed PHQ-9 severity scores of 10 or higher, indicating moderately or severely elevated depression levels. With regard to anxiety, this applies to 23% of the participants (95%CI [18.54%, 28.06%]). Compared to norm samples (comparison studies: depression: [38], anxiety: [27]), participants showed significantly higher depressive and anxiety sum scores in the PHQ-9 (*M*(*SD*) = 9.10(6.0) vs. 2.91(3.52), Cohens *d* = 1.77) and GAD-7 (*M*(*SD*) = 6.51(5.5) vs. 2.95(3.41), Cohens *d* = 1.06). Women showed significantly higher symptomatology compared to men with regard to depression (PHQ-9: *M*(*SD*) = 9.72(6.12) vs. 6.33(4.34), Cohens *d* = .78) as well as anxiety (GAD-7: *M*(*SD*) = 6.92(5.52) vs. 4.67(4.19), Cohens *d* = .54). Depression and anxiety showed a significant positive relationship (*r* = .75, *p* < .01).

### Aspects associated with depression and anxiety

Due to group sizes smaller than *n* = 10, the following ICD-10 chapters were excluded from the regression analysis: Certain infectious and parasitic diseases, diseases of the blood and blood-forming organs and certain disorders involving the immune mechanism, diseases of the eye and adnexa, diseases of the respiratory system, and diseases of the genitourinary system. This results in *n* = 289 participants included in the regression analysis.

#### Depression

All assumptions for linear regression analysis were met. The first block of the regression model, which included gender, age and anxiety as control variables, explained 59% of the variance in depression. Gender and anxiety were significant control variables. Adding the second block with diagnosis-related characteristics (diagnosis assigned to ICD-10 chapter, time since diagnosis, visibility of the symptoms, comorbid diseases) did not contribute significantly to explained variation (Δ*R*^2^ = .02, *F*(10, 275) = 1.38, *p* = .19). None of the newly added predictors were significant. Adding the third block (perceived somatic symptom severity, consequences, personal control, identity, concern, understanding, treatment control, constructive attitudes, active engagement in life, social support) significantly increased the amount of explained variation by 16% (Δ*R*^2^ = .16, *F*(10, 265) = 18.48, *p* < .001). Variables significantly associated with depression after controlling for gender, age and anxiety were perceived somatic symptom severity (*B* = 0.41, *p* < .001), personal control (*B* = 0.18, *p* < .05, higher values indicate less control), concern (*B* = -0.32, *p* < .01) and constructive attitudes (*B* = -1.40, *p* < .001). Table 2 shows results of the regression analysis in detail.

**Table 2:** Hierarchical linear regression model (*n* = 289) with depression as dependent variable.

	Block 1 (control variables)				Block 2 (diagnosis-related aspects)				Block 3 (psychosocial aspects)		
	*B*	*SE(B)*	*ß*		*B*	*SE(B)*	*ß*		*B*	*SE(B)*	*ß*
Intercept	2.64**	0.98			2.47*	1.18			5.70**	1.69	
Gender^1^	1.54*	0.60	0.10		1.46*	0.61	0.10		0.16	0.50	0.01
Age	-0.01	0.02	-0.01		0.00	0.02	-0.01		-0.01	0.02	-0.03
Anxiety	0.82***	0.04	0.74		0.83***	0.04	0.75		0.50***	0.05	0.45
Neoplasms^2^					-2.40	1.43	-0.07		-0.96	1.15	-0.03
Endocrine diseases^2^					-0.48	0.98	-0.02		-0.26	0.79	-0.01
Diseases of the nervous system^2^					0.81	0.90	0.04		1.14	0.74	0.06
Diseases of circulatory system^2^					-0.62	0.95	-0.03		-0.75	0.78	-0.04
Diseases of the digestive system^2^					0.67	0.83	0.05		1.28	0.67	0.09
Diseases of the skin ^2^					-1.28	1.28	-0.04		-0.53	1.01	-0.02
Diseases of the musculoskeletal system ^2^					1.10	1.26	0.04		1.28	1.01	0.04
Time since diagnosis					0.00	0.00	-0.05		0.00	0.00	0.00
Visibility					0.25	0.53	0.02		0.15	0.43	0.01
Comorbid diseases					0.34	0.52	0.03		-0.51	0.42	-0.04
Perceived somatic symptom severity									0.41***	0.05	0.39
Consequences									0.29	0.15	0.09
Personal control^3^									0.18*	0.08	0.08
Identity									-0.07	0.14	-0.03
Concern									-0.32**	0.09	-0.14
Understanding									0.09	0.08	0.04
Treatment control									-0.04	0.07	-0.02
Active engagement in life									-0.21	0.30	-0.03
Constructive attitudes									-1.39***	0.34	-0.21
Social support									-0.08	0.23	-0.02
*R*^*2*^	.59				.61				.77		
Δ *R*^*2*^	.59				.02				.16		
*F* (*df*1, *df*2)	135.31*** (3, 285)				1.38 (10, 275)				18.48*** (10, 265)		

*n* = 289

*p < .05,

**p < .01,

***p < .001, significant results in bold letters.

^1^Reference category: male gender.

^2^Dummy coded variable ICD-10 chapter, reference category: congenital malformations, deformations and chromosomal abnormalities.

^3^higher values indicate less control.

#### Anxiety

Due to heteroscedasticity, we calculated bootstrap confidence intervals for each predictor and their significance values. The first model with gender, age and depression as predictors explained 58% of the variance in anxiety. The second block (diagnosis-related aspects) of the model did not explain significantly more variation (Δ*R*^2^ = .03, *F*(10, 275) = 1.71, *p* = .08) whereas the third block with psychosocial aspects as predictors increased the amount of explained variation by 6% (Δ*R*^2^ = .06, *F*(10, 266) = 4.74, *p* < .000). After controlling for gender, age and depression, diseases of the circulatory system were significantly associated with anxiety (*B* = 1.94, *p* < .05) compared to the reference category (congenital malformations, deformations and chromosomal abnormalities). Further, consequences of the disease (*B* = -0.32, *p* < .05) and concern about the disease (*B* = 0.57, *p* < .01) were significantly related to anxiety. The results of the analysis are displayed in detail in Table 3.

**Table 3:** Hierarchical linear regression model (*n* = 289) with anxiety as dependent variable with 95% bias corrected and accelerated confidence intervals based on 1000 bootstrap samples.

	Block 1 (control variables)				Block 2 (diagnosis-related aspects)				Block 3 (psychosocial aspects)		
	*B*	*SE(B)*	BCa 95% CI		*B*	*SE(B)*	BCa 95% CI		B	*SE(B)*	BCa 95% CI
Intercept	0.59	0.82	[-0.99, 2.29]		0.48	0.96	[-1.45, 2.47]		-3.88*	1.81	[-7.92, 0.05]
Gender^1^	-0.05	0.44	[-0.97, 0.78]		-0.04	0.46	[-0.95, 0.86]		-0.17	0.43	[-1.00, 0.70]
Age	-0.01	0.02	[-0.04, 0.03]		-0.01	0.02	[-0.04, 0.03]		-0.01	0.02	[-0.04, 0.02]
Depression	0.69**	0.04	[0.61, 0.75]		0.69**	0.04	[0.62, 0.75]		0.60**	0.06	[0.48, 0.70]
Neoplasms^2^					1.39	2.04	[-1.97, 5.67]		1.95	1.77	[-1.18, 5.28]
Endocrine diseases^2^					0.87	0.97	[-1.02, 2.76]		1.23	0.96	[-0.66, 3.12]
Diseases of the nervous system^2^					-0.77	0.90	[-2.56, 0.86]		0.00	0.86	[-1.76, 1.64]
Diseases of circulatory system^2^					1.57	0.90	[-0.19, 3.32]		1.94*	0.84	[0.28, 3.51]
Diseases of the digestive system^2^					-0.82	0.73	[-2.32, 0.66]		0.13	0.72	[-1.18, 1.45]
Diseases of the skin^2^					1.29	1.09	[-0.85, 3.48]		1.76	0.99	[-0.16, 3.87]
Diseases of the musculoskeletal system^2^					0.01	1.14	[-2.27, 2.22]		0.21	1.08	[-2.02, 2.25]
Time since diagnosis					0.00	0.00	[0.00,0.01]		0.00	0.00	[0.00, 0.01]
Visibility					-0.09	0.44	[-0.93, 0.77]		0.06	0.43	[-0.75, 0.83]
Comorbid diseases					-0.04	0.47	[-0.93, 0.83]		0.01	0.46	[-0.90, 0.90]
Perceived somatic symptom severity									0.04	0.06	[-0.08, 0.17]
Consequences									-0.32*	0.16	[-0.64, -0.04]
Personal Control^3^									0.06	0.08	[-0.09, 0.21]
Identity									0.13	0.15	[-0.14, 0.44]
Concern									0.57**	0.10	[0.37, 0.75]
Understanding									0.11	0.10	[-0.09, 0.29]
Treatment control									-0.01	0.08	[-0.16, 0.15]
Active engagement in life									-0.57	0.39	[-1.35, 0.15]
Constructive attitudes									0.49	0.43	[-0.27, 1.26]
Social support									0.48	0.27	[-0.05, 1.02]
*R*^*2*^	.58				.60				.66		
Δ *R*^*2*^	.58				.03				.06		
*F* (*df*1,*df*2)	130.61*** (3,285)				1.71 (10,275)				4.74*** (10,265)		

*n* = 289,

*p < .05,

**p < .01,

***p < .001; significant results in bold letters.

^1^Reference category: male gender.

^2^Dummy coded variable ICD-10 chapter, reference category: congenital malformations, deformations and chromosomal abnormalities.

^3^higher values indicate less control.

## Discussion

To the best of our knowledge, this is the first study empirically investigating the frequency of as well as aspects associated with depression and anxiety in patients with different rare chronic diseases. High percentages of the patients showed a clinically relevant degree of symptom burden, reporting at least moderately or severely elevated levels in both depression (42%) and anxiety (23%). This is in line with previous studies on depression and anxiety in more common chronic diseases [3] and empirically supports the high psychological burden of rare diseases stressed by stakeholders such as patient organisations [4, 5]. The high correlation between depression and anxiety is in line with existing research showing that depression and anxiety disorders are frequently co-occurring [39, 40].

The diagnosis-related aspects we took into account were mainly not associated with psychopathology symptoms. The diagnosis assigned to ICD-10 categories was not related to depression and anxiety with one exception: patients with diseases of the circulatory system reported more anxiety compared to patients with congenital malformations. The latter is in line with the particularly high prevalence for anxiety in heart diseases detected in other studies [3]. A meta-analysis showed a consistent association of anxiety disorders with an elevated risk of different cardiovascular diseases [41]. Based on the current cross-sectional data, it is unclear whether increased anxiety symptoms in rare diseases of the circulatory system are a consequence of the somatic symptoms or a risk factor. However, the latter seems unlikely considering that 80% of all rare diseases have a genetic origin [1]. We found no other diagnosis-related differences. This may be surprising since previous studies reported differences in adjustment between various more common chronic conditions [16] and research on specific rare diseases shows different patterns with regard to psychopathology as well [6–8]. However, the evidence on disease-specific differences in more common chronic conditions was mainly derived by studies directly comparing specific diagnoses, which we did not do due to the large number of different rare diagnoses represented in our sample and small sizes of subgroups. The previous evidence on specific rare diseases [6–8] originates from isolated studies with different designs, which therefore can hardly be compared directly. More research is needed to better understand the underlying aspects causing the differences in different chronic conditions and to investigate to what extent this applies to rare chronic diseases. Besides the diagnosis assigned to ICD-10 chapter, neither the time since diagnosis nor the presence of comorbid diseases or visibility of the symptoms were related to psychopathology outcomes. Regarding the visibility of symptoms, these results may be surprising since visibility of disability often goes along with experiences of stigmatization and discrimination and can impact the self-concept [42]. However, qualitative studies with chronically ill patients have shown that the invisibility of a disease can be particularly straining due to disbelief and a lack of understanding by others [43, 44]. This points in the direction that visibility and invisibility of a disease both can be challenging and therefore may lead to increased psychopathology, but due to different reasons. If so, this could explain why we did not find an effect of visibility in the current study.

In summary, the diagnosis-related characteristics we took into account were not associated with psychopathology except patients with rare diseases of the circulatory system showing higher levels of anxiety. In both regression models, the block including all diagnosis-related aspects (diagnosis assigned to ICD-10 chapter, visibility of symptoms, time since diagnosis, comorbid diseases) did not contribute to explained variation. This indicates that investigating psychopathology in patients with different rare conditions concurrently may be a valid approach.

While the diagnosis-related characteristics we considered were mainly not associated with psychopathology outcomes, the subjective perception of and attitudes towards the illness were more relevant. Aspects associated with a higher degree of symptom burden in depression were more perceived somatic symptom severity, less control over the disease, lower levels of concern and less constructive attitudes. The positive association between depression and anxiety and perceived somatic symptom severity replicates findings from other studies, for instance in primary care settings [45] and patients with pain [46]. The same applies to the association between depression and perceived personal control. Illness controllability has been shown to predict good adjustment to a chronic condition [13]. In a systematic review analysing qualitative studies with rare disease patients, control has been identified as a key aspect for successful adjustment, too [19]. Moreover, results of a systematic review on quality of life in patients with rare diseases indicate a strong association between subjective control over the illness and psychological quality of life [47]. The construct of constructive attitudes and approaches represents one facet of cognitive coping-mechanisms and can be described as an attempt to minimize the effects of one’s illness and to not let the illness control their lives [35]. This result is in line with personal control over the disease being positively associated with depression. In the context of psychological adjustment to chronic illness, cognitive appraisal processes are seen as a crucial determinant of disease-related adjustment [15]. In our sample with patients with rare chronic diseases, the association between depression and cognitive appraisal could be replicated. Depression was further associated with less concern, which may seem counterintuitive. In contrast, anxiety was positively associated with concern. The latter is not surprising since concern is a key symptom of anxiety disorders, particularly of generalized anxiety disorders [48]. Since concern seems strongly related to anxiety, the negative association between depression and concern may be a result from statistically controlling for anxiety. Anxiety was further negatively associated with consequences of the disease, which can be understood as the extent of the illness affecting one’s life.

This study has some limitations. It is important to mention that the sample is a convenience sample, which limits the generalizability for all patients with rare diseases. However, epidemiological studies in the field of rare diseases are difficult to conduct due to the high number of different diagnoses and the low prevalence of every single rare disease. In our sample, female and male patients as well as different rare diseases were unevenly distributed with some diagnoses such as neurofibromatosis or primary biliary cholangitis being more frequently represented than others. This may be partly due to our recruitment strategy. We recruited via specialized outpatient clinics and patient organisations. Consequently, we likely reached primarily patients with rare diseases who are relatively well connected to care centres and patients organizations and for whom specialized treatment options are available. It is conceivable that patients with very rare diseases, who may be even more burdened, were not reached by our recruitment process. The high percentage of women in our sample may be the result of a self-selection bias. Men show reduced help-seeking behaviour with regard to mental health problems [49]. Similarly, they may also be less likely to participate in a voluntary survey on mental health. A further limitation is the reliance on self-report regarding relevant variables such as depression and anxiety or the rare disease diagnosis. Particularly concerning the primary outcomes, diagnostic interviews may be more accurate in detecting mood and anxiety disorders. However, both the PHQ-9 and the GAD-7 have shown to be useful and reliable screening tools for depression [21–24] and anxiety [26–28], respectively. Moreover, it is unclear whether the assignment to ICD-10 categories constitutes an accurate representation of disease characteristics. Diseases within one category may differ while others assigned to different categories may have more in common. For instance, rare congenital heart defects are assigned to congenital malformations but might also have aspects in common with diseases of the circulatory system. This limitation reflects a general challenge in research on patients with rare diseases. Directly comparing different rare diseases would mean not to consider a large number of the 7000 different rare diseases. Approaches to build subgroups of different rare diseases can enable researchers to investigate different rare diseases concurrently. The assignment to ICD-10 chapters relies on an internationally accepted system to categorize different diseases based on aetiology and organ system and seemed to be the most standardized and objective option available. But, the fact that we did not find differences between the groups does not allow to reject the difference hypothesis. Directly comparing specific rare diseases or other approaches to build sub-groups may have revealed disease-related differences we did not find. Other approaches to categorize different rare diseases could be evaluated in future research. A statistical limitation concerning the ICD-10 chapters relates to transforming the categorical variable into dummy variables. Congenital malformations served as reference category since this was the largest subgroup. We therefore compared every group with this category but did not make pairwise comparisons between all groups. This is a recommended approach [36, 37] and sufficed in order to estimate the overall association between the ICD-10 diagnosis and depression or anxiety symptoms. However, it remains unclear if other groups may differ from each other regarding the outcomes. Another limitation arises from the cross-sectional design. All effects found in the study display associations and statements about causal relations are not eligible.

Despite these limitations, the current study provides a first empirical insight into psychopathology of patients with different rare chronic diseases and therefore contributes to a relatively unexplored research area. Our results indicate that patients with rare diseases frequently experience symptoms of depression and anxiety underlining the psychological strain that living with a rare disease can cause. The associations with depression and anxiety we found are partly in line with previous research on more common chronic conditions. It remains to be shown by future studies if these findings can be replicated for other rare diseases as well. It further remains unclear which role the rarity of the conditions plays in the development of depression and anxiety. Future research could help better understand the differences between rare and more common chronic diseases.

## Conclusion and clinical implications

This study provides a first insight into depression and anxiety and associated variables in a heterogeneous rare disease sample. Our results show that a high percentage of the patients experience depression and anxiety to a clinically relevant degree.

Mental health and physical health are often closely related. Depression and anxiety in addition to a chronic illness can negatively affect the course of the chronic illness, increase morbidity and mortality rates as well as costs of illness and is a threat to patients’ overall quality of life [9–11]. Detecting and treating depression and anxiety in patients with rare chronic diseases therefore helps improve patients’ overall health. In light of this, systematically screening rare disease patients for depression and anxiety across health care settings may be adequate. In a study with cardiac patients, depression screening combined with written patient-targeted feedback significantly improved depression severity six months after screening [50]. Useful and validated screening tools are available for both depression (e.g. PHQ-9 [21–24]) and anxiety (e.g. GAD-7 [26–28]). Given that 80% of all rare diseases cannot be cured due to their genetic origin [1], effectively addressing depression and anxiety may be one of the few ways in which we can improve patients’ quality of life.

Understanding the conditions under which depression and anxiety emerge in patients with rare diseases helps provide adequate and targeted treatment and prevention offers. In depression, perceived somatic symptom severity as well as cognitive appraisal such as perceived control are associated with symptom degree. In rare disease care, approaches such as shared-decision-making [51] can be particularly useful as they may have the potential to enhance patients’ feelings of control. In anxiety, concern is associated with higher levels of symptomatology. Furthermore, patients with rare diseases of the circulatory system seem to be particularly vulnerable regarding the development of anxiety symptoms. Since anxiety disorders affect overall quality of life and can additionally worsen the course of illness and increase mortality risk in heart diseases [41], clinicians should attempt to rapidly detect and treat anxiety in this patient group. Future research can help better understand the relationship between anxiety and heart diseases in order to adequately address the needs of these patients. No other diagnosis-related aspects were associated with depression or anxiety. This result is limited to the disease-related characteristics we took into account (the diagnosis assigned to ICD-10 chapter, the time since diagnosis, the visibility of the symptoms and comorbid diseases). Nevertheless, our findings indicate that investigating psychopathology in heterogeneous rare disease samples may be appropriate in order to assess shared aspects of different rare diseases. Moreover, this stresses the potential of treatment approaches targeting shared needs of patients with different rare conditions. Subjective perceptions of and attitudes towards the diseases seem to be more relevant than diagnosis-related aspects. Supporting patients in developing adaptive ways to cope with their disease may help reduce psychopathology and therefore improve overall health.
